# Expression analysis of LC3B and p62 indicates intact activated autophagy is associated with an unfavorable prognosis in colon cancer

**DOI:** 10.18632/oncotarget.17554

**Published:** 2017-05-02

**Authors:** Monique Niklaus, Olivia Adams, Sabina Berezowska, Inti Zlobec, Franziska Graber, Julia Slotta-Huspenina, Ulrich Nitsche, Robert Rosenberg, Mario P. Tschan, Rupert Langer

**Affiliations:** ^1^ Institute of Pathology, University of Bern, CH-3008 Bern, Switzerland; ^2^ Graduate School for Cellular and Biomedical Sciences, University of Bern, CH-3008 Bern, Switzerland; ^3^ Institute of Pathology, Technische Universität München, D-81675 München, Germany; ^4^ Department of Surgery, Technische Universität München, D-81675 München, Germany; ^5^ Department of Surgery, Kantonsspital Liestal, CH-4410 Liestal, Switzerland

**Keywords:** colon cancer, autophagy, LC3, p62, immunohistochemistry

## Abstract

Autophagy is a lysosomal degradation and recycling process implicated in cancer progression and therapy resistance. We assessed the impact of basal autophagy in colon cancer (CC) *in vitro* and *ex vivo*. Functional autophagy was demonstrated in CC cell lines (LoVo; HT-29) showing a dose-dependent increase of the autophagy markers LC3B, p62 and autophagic vesciles upon increasing concentrations of the autophagy inhibitor chloroquine, which was demonstrated by immunoblotting, immunofluorescence and electron microscopy. Next, tissue microarrays with 292 primary resected CC, with cores from different tumor regions, and normal mucosa were analyzed by immunohistochemistry for LC3B and p62. CC tissue showed LC3B dot-like, p62 dot-like, cytoplasmic and nuclear staining in various levels without significant intratumoral heterogeneity. Tumoral LC3B and p62 expression was significantly higher than in normal tissue (p<0.001). No associations between staining patterns and pathological features (e.g. TNM categories; grading) were observed. Both low LC3B dot-like and low p62 dot-like-cytoplasmic staining were associated with worse overall survival (p=0.005 and p=0.002). The best prognostic discrimination, however, was seen for a combination of LC3B dot-like/p62 dot-like-cytoplasmic staining: high expression of both markers, indicative of impaired activated autophagy, was associated with the best overall survival. In contrast, high LC3B dot-like/low p62 dot-like-cytoplasmic expression, indicative of intact activated autophagy, was associated with the worst outcome (p<0.001 in univariate and HR=0.751; CI=0.607-0.928; p=0.008 in multivariate analysis). These specific expression patterns of LC3B and p62 pointing to different states of autophagy associated with diverging clinical outcomes highlighte the potential significance of basal autophagy in CC biology.

## INTRODUCTION

Colon Cancer (CC) is a malignancy with one of the highest incidences and is a major cause of cancer-related death worldwide [1, 2]. Individual CC treatment modality is based on tumor localization, tumor extent and biology, as well as additional patient specific factors. In the past several decades this strategy, compromising refined surgerical techniques in combination with adjuvant chemotherapy, highly improved the outcome for early and locally advanced disease. However, CC morbidity and mortality is still generally high [3, 4]. Future medical oncology directives will focus on antitumor treatment that is targeting biological or molecular features of cancer. Autophagy is a cellular catabolic mechanism for the degradation of cellular components via the lysosomal pathway. Ultrastructurally and functionally autophagy is characterized by the formation of double-membraned vesicles called autophagosomes. Autophagy is initiated via the formation of a phagophore or nucleation membrane to which cytoplasmic content is targeted. The phagophore is elongated, resulting in a nascent autophagosome, which engulfs material marked for degradation and closes. Finally, mature autophagosomes fuses with lysosomes, resulting in autolysosomes, and content is degraded via catalytic enzymes. Sugars, fatty acids, amino acids and nucleosides are thus released back into the cytoplasm. Physiological functions of autophagy include maintainance of energy homeostasis, elimination of defective organelles and proteins, prevention of reactive oxygen species and removal of intracellular pathogens. Dysfunction of autophagy is associated with autoimmune, cardiac and neurodegenerative diseases, as well as cancer. In healthy cells, autophagy maintains normal metabolism, prevents inflammation, oxidative stress and DNA damage by removing damaged organelles and proteins. It is therefore theorized that in early stages of cancer, autophagy has tumor-suppressing properties. However, in late stages it is thought that autophagy allows survival, dormancy, growth and metastasis by providing an alternative energy source therefore exhibiting tumor-promoting properties [5, 6]. Two widely used autophagy markers are microtubule-associated protein 1 light chain 3 B (LC3B) and p62/sequestosome 1 (SQSTM1). During autophagy the cytosolic LC3B-I form is converted into the lipidated LC3B-II form associated with autophagosomal membranes. p62 targets ubiquitinated substrats to autophagosomes via its interacts with LC3B. During autophagic flux both proteins are subject to degradation in autolysosomes [7, 8]. Several reports point to a role of autophagy in CC biology and treatment response. We aimed to investigate the impact of autophagy under basal conditions *in vitro* in CC cell lines and *ex vivo* in CC tumor tissue.

## RESULTS

### Investigation of intact functional basal autophagy of CC cells *in vitro*

**Figure 1:**
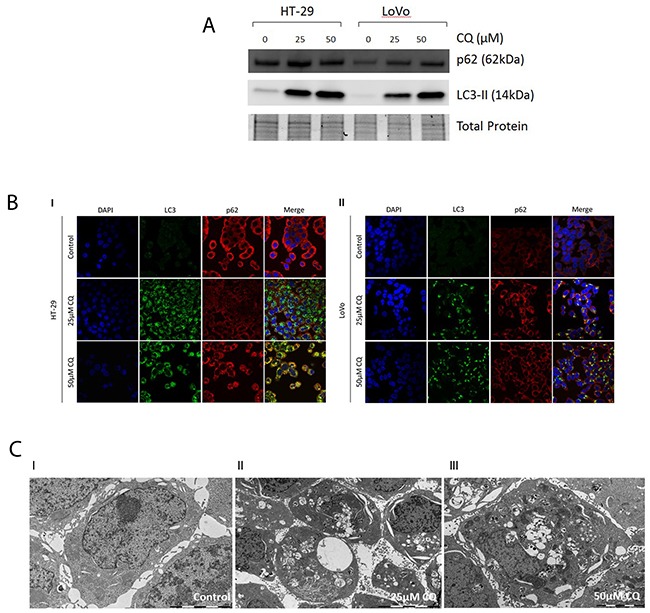
Assesment of functional basal autophagy *in vitro* **(A)** Immunoblotting of LC3B and p62 upon pharmacological autophagy inhibition. A dose-dependent increase of LC3B and p62 was observed with increasing concentrations of chloroquine. **(B)** Immunofluorescence of LC3B and p62 upon pharmacological autophagy inhibition. I. In CC cell lines HT-29 and II. LoVo a dose-dependent increase of LC3B and p62 was observed with increasing concentrations of chloroquine. **(C)** Electron microscopy of CC cell lines pharmacological autophagy inhibition. I-III. In CC cell line HT-29 a dose-dependent increase of cytoplasmic vesicles was observed with increasing concentrations of CQ.

The expression of the autophagy markers LC3B and p62 were assessed via immunofluorescence and immunoblotting after pharmacological autophagy inhibition with chloroquine, which prevents the fusion of autophagosomes and lysosomes by increasing lysosomal pH, in HT-29 and LoVo CC cell lines. Additionally, we visualized autophagic vacuoles via electron microscopy after chloroquine treatment. We found a dose-dependet increase of both autophagy markers LC3B and p62 with increasing concentrations of chloroquine as assessed by immunoblotting and immunofluorescence (Figure 1A and 1B). These results are indicative of functional basal autophagy in both cell lines. Electron microscopy results showed an increase in the formation of cytoplasmic vacuoles, often engulfing cellular components or organelles (Figure 1C). Although the typical double membraned characteristic could not be visualized, these structures can be interpreted as being autophagosomes as the decreased autophagosome degradation upon pharmacological autophagy inhibition can serve as plausible explanation for the presence of the abundant vesicles observed. Together, our data clearly indicate that both cell lines display functional basal autophagy.

### Patho-clinical and prognostic implications of the expression of autophagy markers in CC tissue

**Figure 2:**
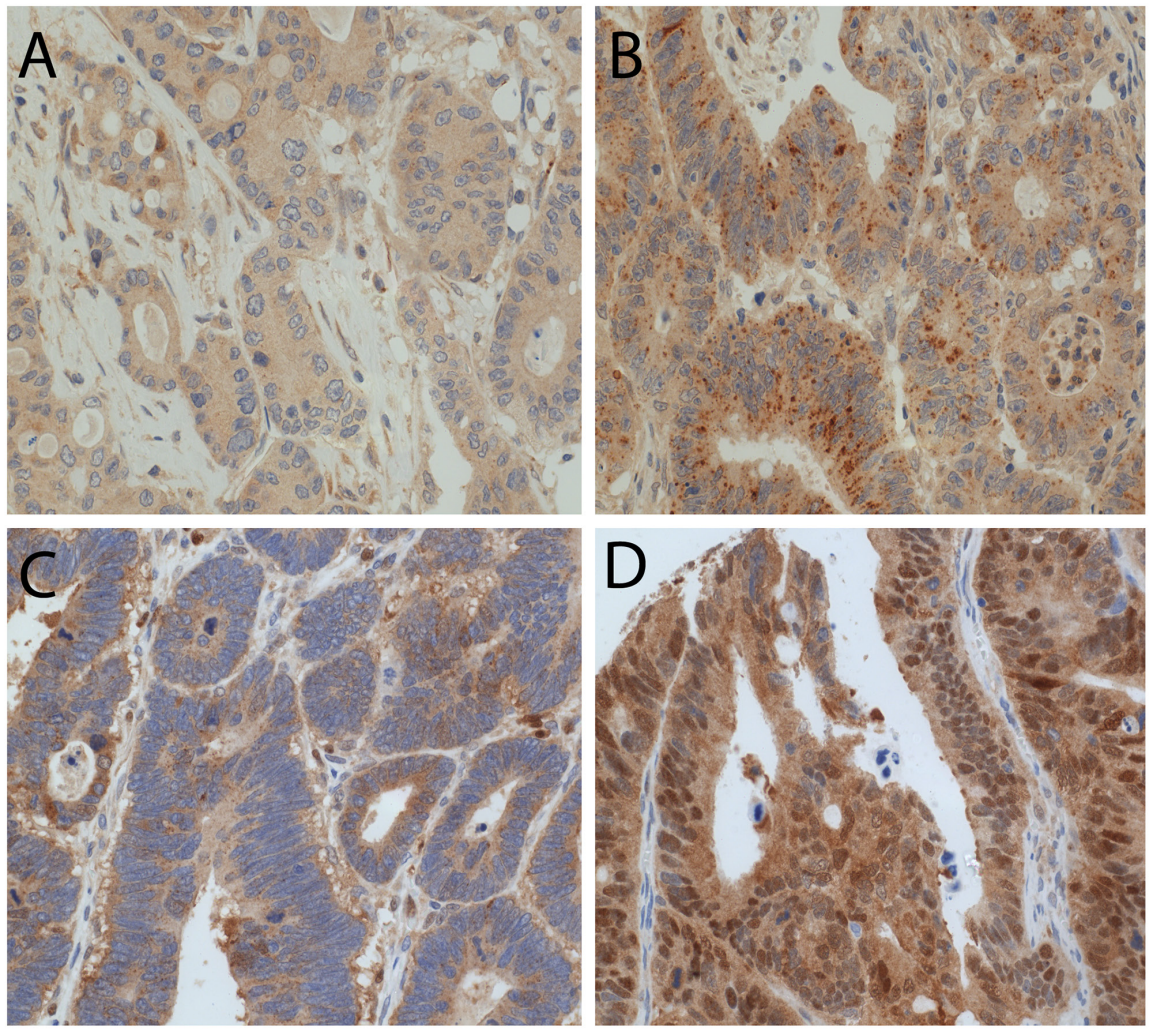
LC3B and p62 immunohistochemical staining in CC tissue **(A)** CC with low LC3B dot like staining. **(B)** CC with high LC3B dot like staining. **(C)** CC with both high p62 dot like and high p62 cytoplasmic staining. **(D)** CC with positive p62 nuclear staining (magnification 20X).

**Figure 3:**
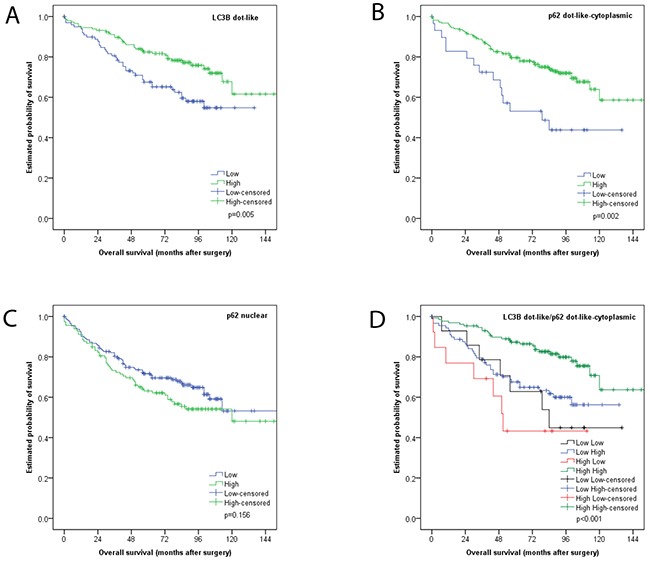
Kaplan Meier survival analysis for LC3B and p62 immunohistochemical staining in CC tissue **(A)** LC3B dot like staining **(B)** p62 dot like-cytoplasmic staining **(C)** p62 nuclear staining **(D)** combination of LC3B dot like/p62 dot like-cytoplasmic staining.

We observed LC3B dot-like and p62 dot-like immunohistochemical staining was observed ranging from absent (score 0) to strong (score 3) in CC tissue and non-neoplastic colon mucosa. Additionally a range of p62 cytoplasmic as well as both negative and positive p62 nuclear immunohistochemical staining was reported (Figure 2). LC3B dot-like and p62 dot-like immunohistochemical staining showed a positive association (p<0.001). A positive association was also seen between p62 dot-like and p62 cytoplasmic immunohistochemical staining (p<0.001). Tumor tissue from the tumor center and tumor periphery showed significantly higher amounts of LC3B dot-like, p62 dot-like, p62 cytoplasmic and p62 nuclear immunohistochemical staining compared to normal non-neoplastic colon mucosa (p<0.001). There was no significant intratumoral heterogeneity with comparable staining patterns for all markers in the tumor center and the tumor periphery. No significant associations with staining patterns and pathoclinical features, such as pT category, presence of lymph node metastasis or distant metastasis, grading, as well as tumor location and MMR status were observed (Tables 1, 2 and 3). Survival analysis demonstrated that both low LC3B dot-like and low p62 dot-like-cytoplasmic stainings were associated with a worse overall survival, in univariate (p=0.005 for LC3B and p=0.002 for p62) and multivariate analysis (HR=0.599; CI=0.376-0.954; p=0.031 for LC3B and HR=0.507; CI=0.285-0.902; p=0.021 for p62). In addition, the combination of LC3B dot-like and p62 dot-like-cytoplasmic staining showed the best prognostic discrimination in univariate (p<0.001) and multivariate analysis (HR=0.751; CI=0.607-0.982; p=0.008). High expression of both markers, indicative of impaired activated autophagy, was associated with the best prognosis in contrast to high LC3B dot-like/low p62 dot-like-cytoplasmic, indicative of intact activated autophagy, staining with the worst prognosis (Table 4; Figure 3).

**Table 1:** Correlation of LC3B dot-like immunohistochemical staining with patho-clinical features

Parameter		N	LC3B dot-like low	LC3B dot-like high	p-Value
**pT category**	pT1	11	5	6	0.845
	pT2	50	18	32	
	pT3	160	67	93	
	pT4	71	27	44	
**Lymph node metastasis**	Absent	180	67	113	0.359
	Present	111	50	61	
**Distant metastasis**	Absent	252	102	150	0.695
	Present	39	15	24	
**Grading**	G1-G2	195	83	112	0.169
	G3-G4	97	34	63	
**Resection status**	R0	257	103	154	0.832
	R1	35	14	21	
**Location**	Right	53	24	29	0.392
	Left	239	93	146	
**MMR status**	Proficient	258	101	157	0.376
	Deficient	34	16	18	

**Table 2:** Correlation of p62 dot-like-cytoplasmic immunohistochemical staining with pathoclinical features

Parameter		N	p62 dot-like/cytoplasmic low	p62 dot-like/cytoplasmic high	p-Value
**pT category**	pT1	11	1	10	0.209
	pT2	50	5	45	
	pT3	160	19	141	
	pT4	71	15	56	
**Lymph node metastasis**	Absent	180	21	159	0.150
	Present	111	19	92	
**Distant metastasis**	Absent	252	30	222	0.062
	Present	39	10	29	
**Grading**	G1-G2	195	28	167	0.635
	G3-G4	97	12	85	
**Resection status**	R0	257	31	226	**0.004**
	R1	35	9	26	
**Location**	Right	53	9	44	0.442
	Left	239	31	208	
**MMR status**	Proficient	258	32	226	0.076
	Deficient	34	8	26	

**Table 3:** Correlation of p62 nuclear immunohistochemical staining with pathoclinical features

Parameter		N	p62 nuclear low	p62 nuclear high	p-Value
**pT category**	pT1	11	8	3	0.513
	pT2	50	34	16	
	pT3	160	94	66	
	pT4	71	41	30	
**Lymph node metastasis**	Absent	180	119	61	0.069
	Present	111	58	53	
**Distant metastasis**	Absent	252	152	100	0.417
	Present	39	25	14	
**Grading**	G1-G2	195	119	76	0.497
	G3-G4	97	58	39	
**Resection status**	R0	257	155	102	0.861
	R1	35	22	13	
**Location**	Right	53	35	18	0.372
	Left	239	142	97	
**MMR status**	Proficient	258	158	100	0.548
	Deficient	34	19	15	

**Table 4:** Multivariate analyses

**A**				
**Parameter**	**HR**	**95% confidence interval**		**p-Value**
		**min**	**max**	
**pT category**	1.397	0.98	1.99	0.064
**Lymph node metastasis**	1.942	1.199	3.147	**0.007**
**Grading**	1.046	0.634	1.724	0.86
**Age**	4.093	2.418	6.929	**0.001**
**LC3B dot-like staining**	0.599	0.376	0.954	**0.031**
**B**				
**Parameter**	**HR**	**95% confidence interval**		**p-Value**
		**min**	**max**	
**pT category**	1.355	0.95	1.933	0.094
**Lymph node metastasis**	2.088	1.284	3.396	**0.003**
**Grading**	1.071	0.645	1.777	0.792
**Age**	4.084	2.409	6.921	**0.001**
**p62 dot-like/cytoplasmic**	0.507	0.285	0.902	**0.021**
**C**				
**Parameter**	**HR**	**95% confidence interval**		**p-Value**
		**min**	**max**	
**pT category**	1.408	0.983	2.016	0.062
**Lymph node metastasis**	1.87	1.151	3.037	**0.011**
**Grading**	1.058	0.641	1.749	0.825
**Age**	4.024	2.374	6.82	**0.001**
**LC3B dot-like/p62 dot-like-cytoplasmic**	0.751	0.607	0.928	**0.008**

A) model including LC3B dot-like immunohistochemical staining; B) model including p62 dot-like-cytoplasmic immunohistochemical staining; C) model including the combination of LC3B dot-like/p62 dot-like-cytoplasmic immunohistochemical staining.

**Figure 4:**
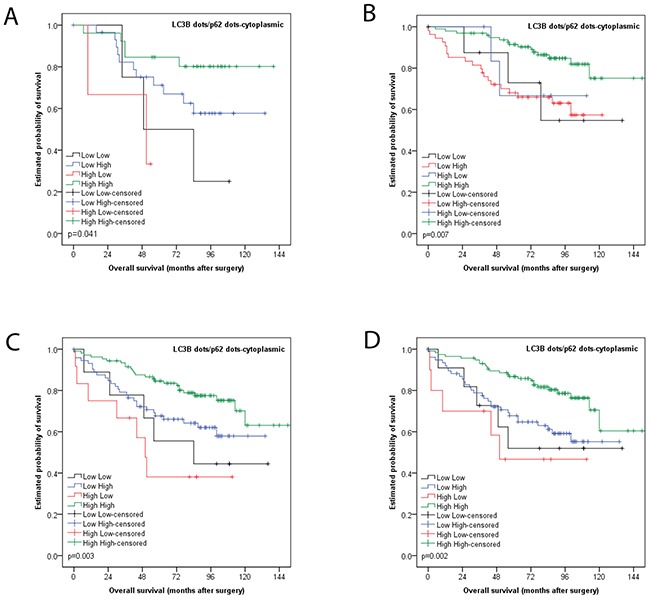
Kaplan Meier survival analysis for combined LC3B dot like/p62 dot like-cytoplasmic staining for subgroups **(A)** Tumors with adjuvant treatment **(B)** non-metastasized tumors (i.e. UICC/AJCC stages I-II) **(C)** left sided carcinomas **(D)** MMR proficient carcinomas

In subgroup analyses, the prognostic impact of this classification was also seen for non-metastasized tumors (i.e. UICC/AJCC stages I-II; p=0.007), tumors with adjuvant treatment (p=0.041), left sided carcinomas (p=0.003) and MMR proficient carcinomas (p=0.002) in univariate analyses (Figure 4).

## DISCUSSION

We investigated the impact of basal autophagy, focusing on two autophagy related proteins, in colon cancer. Immunoblotting and immunofluorescence results showed a dose-dependent increase of both autophagy marker proteins LC3B and p62 with increasing concentrations of autophagy inhibitor chloroquine. Since LC3B and p62 are incorporated into autophagosomes and subsequently degraded in autolysosmes, the accumulation of these proteins upon pharmacological autophagy inhibition is indicative of functional basal autophagy in the two CC cell lines tested [9]. Similarly, electron microscopy results indicated a dose-dependent increase in autophagic vesicles with increasing concentrations of chloroquine, supporting our immunoblotting and immunofluorescence data. Autophagic flux under nutrient starvation and steady state conditions with the use of pharamcological autophagy inhibition and LC3B detection in CC cell lines had been demonstrated before [10]; this is in line with our *in vitro* data, which shows intact autophagic machinery under steady state conditions in CC cell lines.

The current study also highlights the biological and prognostic impact of autophagy related markers LC3B and p62 *ex vivo* in CC tissue. In our cohort tumor tissue exhibited higher expression of both LC3B and p62, as detected via IHC, when compared to adjacent normal colon tissue. Interestingly no significant intratumoral heterogeneity, with respect to LC3B and p62 expression, was observed when comparing tumor center and periphery. When assessing single staining patterns, both low LC3B dot-like and low p62 dot-like-cytoplasmic stainings were associated with a worse outcome. To date several studies have been published which also report higher expression levels of LC3 and p62, together with other autophagy markers, in CC or colorectal cancer (CRC) tissue when compared to normal mucosa [11–13]. In contrast, others have also observed decreased expression of different autophagy associated proteins in CRC [14, 15]. As is the case when comparing autophagy marker expression levels in normal and cancer tissues, so too are the results regarding correlation to pathoclinical features divergent. Most papers report correlations between the expression of autophagy related proteins and the degree of tumor differentiation, but are inconstant with their findings [11–13, 16].

The body of literature also lacks consistancy regarding the prognostic impact and relevance of autophagy markers in CC and CRC. Some groups report that low expression levels of LC3 correlated with a worse prognosis and overall survival in their respective cohorts and studies, which is in line with our findings regarding single staining analysis [11, 14, 16]. Others found that LC3 overexpression correlated with a worse overall survival, in part, however, only in specific molecular defined (i.e. KRAS-mutated) subpopulations [17].

The discrepencies found in the literature can be attributed to various confounding factors. It could be due to genetic differences in the cohorts stemming from different populations around the world. CC and CRC are very heterogeneous diseases and molecular characteristics such as KRAS, BRAF and microsatellite instability has not been used to further stratify cohorts. The fact that IHC staining and scoring protocols for the most commonly used autophagy markers, particularly LC3B, is not standardized, can also be an important confounding factor. Antibody specificity as well as interpretation of staining patterns can result in greatly varying results. Our group previously established and validated IHC staining and scoring protocol for LC3B (and p62) and found that only LC3B punctate staining patterns correlates with autophagy induction as opposed to diffuse cytoplasmic staining. The interobserver agreement was acceptable, especially after training, which made us comfortable to regard our evaluation results as reliable, despite the characteristic dot-like staining pattern that is not easy to score [18]. Few groups, however, distinguish between punctae and diffuse cytoplasmic staining patterns and thereby not discriminating between the LC3B-II isoform which is associated with autophagosomes and the LC3B-I isoform which is not. Standardizing IHC staining and scoring protocols for autophagy should be regarded as necessary steps towards using ATG proteins as biomarkers for research and potentially future routine diagonostic pratice for CC. However, using a snapshot of one protein of a highly dynamic process such as autophagy has its pitfalls, particularly when the markers used are themselves subject to autophagic degradation. Furthermore, single staining analysis gives limited insight into the underlying biological role of autophagy in any given disease setting. Therefore, in the present study we employed an analysis strategy, which involves formulating groups based on both LC3B and p62 staining in order to elucidate an autophagic index for each subpopulation. This approach was first applied on oral squamous cell carcinoma by others, where high cytoplasmic p62 expression accompanied with either a low or high LC3B expression, indicative of autophagy impairment under basal or activated autophagic activity, was associated with aggressive behaviour in advanced tumors [19]. In the current study, we report that the combination of LC3B dot-like/p62 dot-like-cytoplasmic staining showed the best prognostic discrimination. High LC3B dot-like/high p62 dot-like-cytoplasmic staining (indicative of impaired autophagy) was associated with the best prognosis, in contrast to high LC3B dot-like/low p62 dot-like-cytoplasmic staining (indicative of activated autophagy) with the worst prognosis. This is in contrast to the findings of the OSCC study. However, our group subjected an esophageal adenocarcinoma cohort to similar analysis and found that the group with intact basal autophagy faired the worse [20]. The latter study suggests a similar underlying biology with respect to autophagic activity in upper and lower gastrointestinal cancers. Other authors created an autophagic score, compromising of the staining patterns of ATG5, Beclin1 and LC3B, and found that a low autophagic score correlated with a worse prognosis in CRC or demonstrated that a combination of Beclin1, LC3B and Bcl-xL (classically thought of as apoptosis marker) also showed better prognostic discrimination than analysis done on single staining [14, 16]. This underscores the importance of the strategy taken in our current findings. However caution should be used when combining staining patterns of ATG genes, which are players at different stages of the autophagic process and it should be taken into account whether or not these proteins are subject to autophagic degradation and which staining patterns are associated with autophagy induction.

Autophagy has been described as a mechanism for tumors to develop resistance against chemotherapy or other kinds of cytotoxic treatment. Park *et al*. and Guo *et al*. reported on the association between the expression of autophagy markers and response to chemotherapy and targeted therapy against EGFR, respectively [21, 22]. We analyzed the expression of LC3B and p62 in a subset of CC that had been treated with adjuvant therapy after surgery, which was the case for most of the lymph node metastases and in line with treatment recommendations for clinical practice. The prognostic impact of the expression of our markers and the combination, respectively, was comparable to the whole collection, and the direct comparison group of stage I-II tumors that usually do not receive adjuvant treatment. Therefore, we cannot draw significant conclusions regarding the role of tissue expression of LC3B and p62 for chemotherapy reponse, although the number of cases in this particular subgroup was higher than reported from Park *et al*. [21].

In summary, our study highlights the importance of autophagy in CC biology. Given that we observed no significant intratumoral heterogeneity with respect to expression levels of LC3B and p62, and tumors consistently showed higher expression levels when compared to normal mucosa, this strongly suggests that aberrant autophagic regulation and activity is an *a priori* state of the tumor, as opposed to a reactive process. Of note, we observed no difference in prognostic impact in the subgroup treated with adjuvant chemotherapy when compared to treatment naïve patients, which is in contrast to studies done in other tumor entities which report autophagy as a resistance mechanism activated upon cytotoxic treatment. However, given that the subgroups assigned with having activated autophagy exhibit the worse overall survival suggests that autophagy is exploited by the tumor entity as an oncogenic advantage. Elucidating the role of autophagy in CC therefore presents itself as a promising avenue to develop novel, targeted therapeutic strategies which would be feasible given the number of clinical trials involving pharmacological autophagy inhibitors currently ongoing for various tumor types [23–28].

## MATERIALS AND METHODS

### Cell lines, culture and treatment conditions

Human CC cell lines HT-29 and LoVo were obtained from ATCC LGC Standards GmbH, Switzerland. HT-29 cells were maintained and cultered in DMEM Medium (Sigma-Aldrich, D6046), supplemented with 10% fetal bovine serum (Sigma-Aldrich, F7524), 1.25% L-Glutamine 1.25% and 1% penicillin/streptomycin. LoVo cells were cultered in F-12K Medium (Gibco Life Technologies, 21127022) supplemented with 10% fetal bovine serum (Sigma-Aldrich, F7524) and 1% penicillin/streptomycin. Cells were treated with chloroquine (Sigma-Aldrich, C6628) at 25 μM and 50 μM for 24 hours. Subsequently, cells were fixed or harvested for immunofluorescence or immunoblotting, respectively.

### Immunofluorescence

Cells were washed in phosphate buffered saline (PBS), prefixed in 4% paraformaldehyde for 15s at room temperature (RT), fixed in ice-cold 100% methanol for 10min at 20°C and washed PBS at RT. Primary antibodies, namely anti-LC3B antibody (Cell Signaling, Lausen, Switzerland, rabbit monoclonal, clone D11, #3686) and anti-p62 antibody (Sigma, Leiden, Netherlands, mouse monoclonal, clone 2C11, #WH0008878M1) were diluted 1:100 and 1:200 in PBS/1% bovine serum albumin (BSA)/0.1% Tween, respectively. Slides were incubated with primary antibodies for 1h at RT. Slides was washed twice with PBS/0.1% Tween and once with PBS only. Secondary antibodies, namely FITC conjugated anti-rabbit antibody (Jackson Immunoresearch, Suffolk United Kingdom, #111-096-045) and Cy3 conjugated anti-mouse antibody (Jackson Immunoresearch, Suffolk, United Kingdom, #115-166-003), were diluted 1:130 in PBS/1%BSA/0.1%Tween. Slides were incubated with secondary antibodies for 1h at RT. Images were taken on Olympus FluoView microscope at 63x objective magnification and adjusted for brightness using ImageJ Software (NIH, Bethesda, MD, United States of America, 1.64r).

### Immunoblotting

Cells were lysed in a buffer containing urea 8M, tritonX 0.5% and a protease inhibitor (Complete midi, Roche Diagnostics, Rotkreuz, Switzerland). Cells were sonicated and centrifuged at 13’000 rcf for 30min at 4°C. The supernatant was transferred to a fresh tube. Protein concentration was determined using Bradford protein assay (BioRad, Cressier, Switzerland). Protein totalling 30μg was denatured in sample buffer and β-mercaptoethanol at 95°C for 5min. Samples were loaded on a 4-20% stain-free pre-cast gel (BioRad, Cressier, Switzerland) using Mini-Protean® Tetra Vertical Electrophoresis Cell System (BioRad, Cressier, Switzerland) at 200V for 30min. Samples were transferred onto a polyvinylidene difluoride membranes using Trans-Blot® Turbo^TM^ Transfer System (BioRad, Cressier, Switzerland). Membranes were blocked with 5% BSA/TBS for LC3B and 5% milk/TBS for p62 with shaking for 1h at RT. Primary antibodies, namely anti-LC3B antibody (LuBioScience, Luzern, Switzerland, rabbit polyclonal, #NB600-1384) and anti-p62 antibody (Sigma-Aldrich, Leiden, Netherlands, mouse monoclonal, #WH0008878M1), were diluted 1:1000 in 5% milk/TBS/0.1%Tween. Membranes were incubated with primary antibodies with shaking overnight at 4°C. Secondary antibodies, namely DyLight®650 conjugated goat anti-rabbit antibody (LabForce, Switzerland) and DyLight®550 conjugated goat anti-mouse antibody (LabForce, Switzerland), were diluted 1:1000 in 5% milk/TBS. Membranes were incubated with secondary antibodies with shaking for 1h at RT. Total proteins and proteins of interest were acquired and visualized using ChemiDoc^TM^ MP System and Image Lab Software (BioRad, Cressier, Switzerland). Resulting images were adjusted for brightness in ImageJ Software (NIH, Bethesda, MD, United States of America, 1.64r).

### Electron microscopy

HT-29 and LoVo grown and treated as decribed above on inserts (BD Falcon, Milan Dutscher Group, Nesselnbach/Niederwil, Switzerland, #353090) in six-well-plates were processed for electron microscopy as follows: Each insert was fixed in a solution of 3% Glutaraldehyde (25% EM Grade, Agar Scientific, Stansted, UK, AGR1010) in 0.1 M potassium phosphate buffer (total osmolarity 630 mOsm, pH 7.2). Thereafter the inserts were postfixed in a 1% solution of Osmium Tetroxide (Electron Microscopy Sciences, Hatfield, USA) in 0.1 M sodium cacodylate buffer (total osmolarity 356 mOsm, pH 7.2) and dehydrated in increasing concentrations of ethanol, 70%, 80%, 96% and 100%. The membranes of the inserts were cut out and embedded in Epon 812 (Sigma Aldrich, Wien, Austria, 45345-250ML-F). Ultrathin sections of 70 nm were cut in flat angle on a Reichert-Jung Ultracut E Microtome using a diamond knife (Diatome, Biel, Switzerland), put on 200 mesh hexagonal copper grids (Plano, Wetzlar, Germany) and double stained with 1% Uranyl Acetate (Sigma Aldrich, Wien, Austria, 73943) and 3% Lead Citrate (Leica Microsystems, Heerbrugg, Switzerland, 16705530). Electron micrographs were taken on a Morgagni M268 Electron Microscope (FEI, Brno, Czech Republic).

### Patient tissue samples

Formalin fixed and paraffin embedded (FFPE) tissue of colon adenocarcinoma resection specimens of 292 patients was investigated. All patients had been treated by primary resection without neoadjuvant treatment between 1993 and 2005 at Klinikum Rechts der Isar, Technische Universität München, München, Germany, as previously described elsewhere [29, 30]. Median age of the patients was 66 years (ranging from 25 to 91 years), male/female ratio was 160/132 and median survival was 74.5 months (ranging from 0 to 164 months). The tumor was located in right hemicolon in 53 cases (18.2%) and in left hemicolon in 239 cases (81.8%). The pT category (according to UICC 2009) was pT1 in 11 cases (3.8%), pT2 in 50 cases (17.1%), pT3 in 160 cases (54.8%) and pT4 in 71 cases (24.3%). Lymph node metastasis were absent in 180 cases (61.9%) and present in 111 cases (38.1%). Distant metastasis at the time of surgery were absent in 254 cases (87.0%) and present in 38 cases (13.0%). Tumor grading was G1-G2 (well differentiated) in 195 cases (66.8%) and G3-G4 (poorly differentiated) in 97 cases (33.2%). Complete tumor resection was achieved in 257 cases (88.0%). Adjuvant treatment (chemotherapy) was given in 94 cases, of which the majority was stage III cancers. Consent for additional molecular analysis was given by the patient at the time of the surgery. The use of human tissue for additional molecular analysis was approved by the local ethics committee (2136/08).

### Immunohistochemical staining, scoring and subclassification

Three punches (d=0.6mm) were taken from tumor center, tumor periphery and adjacent non-neoplastic colon mucosa to construct a next generation tissue microarray (ngTMA) with digital annotation of scanned slides and automatic transferal of the punches, as previously described [31]. After deparaffination, rehydration and antigen retrieval, immunohistochemical staining was performed on 4μm sections using an automated immunostainer Leica Bond RX (Leica Biosystems, Heerbrugg, Switzerland) as described before [18], as follows: LC3B (Novus Biologicals, Zug, Switzerland, rabbit polyclonal, #NB600-1384): 1:4’000, tris buffer, 95°C, 30min; p62/SQSTM1 (LabForce mbl, Nunningen, Switzerland, rabbit polyclonal, #PM0045): 1:8’000, citrate buffer, 100°C, 30min. Visualization was performed using the Bond Polymer Refine Detection kit (Leica Biosystems, Muttenz, Switzerland, DS9800) according to the manufacturer's instructions. Immunohistochemical stainings were scored across the respective cores of the different tumor regions, and finally for all cores of one tumor according to a previously established protocol which has also been applied in other studies [20, 32]. LC3B and p62 dot-like immunohistochemical staining was scored from 0 to 3 as follows: score 0 - no dots visible or barely dots visible in < 5% of the cells, score 1 - detectable dots in 5-25% of the cells, score 2 - detectable dots in 25-75% of the cells, score 3 - dots visible in > 75% of the cells. p62 cytoplasmic immunohistochemical staining was scored from 0 to 3 as follows: score 0 - no or faint cytoplasmic staining visible, score 1 - weak cytoplasmic staining visible, score 2 - moderate cytoplasmic staining visible and score 3 - strong cytoplasmic staining visible. p62 nuclear immunohistochemical staining was scored from 0 to 1 as follows: score 0 - nuclear staining visible in < 10% of nuclei and score 1 - nuclear staining visible in > 10% of nuclei. Scoring was performed by two observers (MN and RL) on a Zeiss Axioscope microsope at 40x objective magnification. Discrepant results were re-evaluated at a double header microscope. Examples of LC3B and p62 immunohistochemical stainings are shown in Figure 2. For the purpose of correlation with pathoclinical features the immunhistochemical scores were catagorized as either low or high for each staining pattern according to the prognostic impact of the single scores, as described before [20, 32]. For LC3B dot like staining scores 0 and 1 were classified as low and scores 2 and 3 were classified as high. The low category of p62 dot like staining was assigned to score 0, while scores 1, 2 and 3 were assigned to the high category. p62 cytoplasmic staining scores were similarly subdivided. p62 nuclear staining score 0 was classified as low and score 1 was classified as high. A combination score of p62 dot-like-cytoplasmic staining was calculated by adding dot-like and cytoplasmic staining raw scores, with score 0 and 1 being classified as low and scores 2 through 6 being classified as high. In line with a previous study the dataset was also stratified into 4 subtypes: low LC3B dot-like/low p62 dot-like-cytoplasmic staining (LL), low LC3B dot-like/high p62 dot-like-cytoplasmic staining (LH), high LC3B dot-like/low p62 dot-like-cytoplasmic staining (HL) and high LC3B dot-like/high p62 dot-like-cytoplasmic staining (HH) [19]. Immunohistochemistry for mismatch repair (MMR) proteins was performed on the Leica Bond III autostainer as described [33]. Tumors were classified as MMR-proficient if MLH1, MSH2, PMS2 and MSH6 were expressed. MMR-deficiency was defined as absence of the expression of one or more of these markers.

### Statistical analysis

The SPSS 23 software (SPSS Inc, Chicago, USA) was used for descriptive and comparative statistical analysis, for univariate and multivariable analysis, respectively. Associations between immuno-histochemical stainings and pathoclinical features were evaluated using cross tabs (Chi-square-test or Fisher's exact test). Survival analysis was performed using log rank test and Cox regression analysis and was done for patients with complete tumor resection (R0) and without distant metastases (M0). The significance level was set at 0.05.
